# The Effects of a Standardized Cognitive-Behavioural Therapy and an Additional Mindfulness-Based Training on Interoceptive Abilities in a Depressed Cohort

**DOI:** 10.3390/brainsci11101355

**Published:** 2021-10-15

**Authors:** Georgios Karanassios, Dana Schultchen, Matthias Möhrle, Götz Berberich, Olga Pollatos

**Affiliations:** 1 Clinical and Health Psychology, Institute of Psychology and Education, Ulm University, 89069 Ulm, Germany; dana.schultchen@uni-ulm.de (D.S.); matthias.moehrle@uni-ulm.de (M.M.); olga.pollatos@uni-ulm.de (O.P.); 2 Klinikum Windach a. Ammersee, 86949 Windach, Germany; berberich@klinik-windach.de

**Keywords:** interoception, depression, cognitive-behavioural therapy, mindfulness, MBSR

## Abstract

Background: Interoceptive accuracy and sensibility are decreased in depressive samples. However, different studies showed that cognitive-behavioural therapy (CBT) and mindfulness interventions are promising approaches to improve interoceptive abilities. Based on these findings, the study aims to investigate the pre–post effect of CBT in a depressive sample. Additionally, we examined the effect of mindfulness-based stress reduction (MBSR) training in the context of CBT. Methods: Sixty depressive patients were investigated over four weeks, with two conditions—CBT vs. CBT + MBSR. Further, the changes in interoceptive abilities (interoceptive accuracy and sensibility) of the depressive patients were compared to baseline data of healthy controls. Results: The depressive patients showed significantly higher levels of depression and lower mindfulness and interoceptive abilities than healthy controls. The depressive sample showed a significant decrease in depressive symptoms and increased mindfulness and interoceptive abilities after CBT. Lastly, depressive patients of the CBT + MBSR condition did not differ from those who only received CBT in the levels of depression, mindfulness or interoceptive abilities over the time course. Discussion: This study demonstrates a positive effect of CBT on interoceptive abilities in a depressive sample. It is shown that the depressive sample did not profit from additional mindfulness training. It can be concluded that CBT is an efficient treatment, resulting in increased interoceptive abilities. Unexpectedly, the combination of CBT and MBSR has no additional effect on these changes. Future studies should investigate the effect of MBSR as a stand-alone therapy.

## 1. Introduction

Interoception is the ability to perceive and process sensory information arising from within the body [1,2]. It can be described as a three-dimensional construct, including interoceptive accuracy (IAcc), interoceptive sensibility (IS) and interoceptive awareness, which differ mainly in terms of their assessment [3]. In this paper, IAcc and IS are relevant. Consequently, both dimensions will be explained in detail in the following. IAcc can be described as an objective dimension of interoception [4]; more precisely, it refers to the ability to perceive different bodily signals, such as cardiovascular, gastrointestinal, and respiratory signals. IAcc describes the individual performance in a behavioural test. It is described through the degree of agreement with objectively recorded parameters (e.g., heartbeats measured via ECG) and subjective received parameters (e.g., counted heartbeats). In previous research, the heartbeat tracking task was mainly used to assess IAcc. In this task, participants were instructed to count their heartbeat silently during different time intervals. Higher degrees of difference between the recorded heartbeats (via ECG) and the counted heartbeats indicate a higher IAcc. In contrast, IS is based on the beliefs about one’s perception of interoceptive signals (subjective assessment). Typical assessments include different self-reports such as confidence ratings regarding the heartbeat tracking task or the Body Perception Questionnaire (BPQ) [5]. Confidence ratings are assessed directly after the IAcc. Here, participants are asked how confident they feel regarding their counted heartbeats. The BPQ focuses on the daily perception of the bodily signals, symptoms and processes (e.g., “During most situation I am aware of how fast I am breathing”). It can be concluded that IS focuses on cognitive processes such as attention to the body and related ratings, memories, and attitudes. Consequently, the focus of this dimension is based on the subjective assessment of interoceptive perception. Compared to the self-report questionnaires regarding bodily signals in general, the confidence rating is more specific because it is assessed directly after the IAcc task. To summarize, there are methodological differences for the assessment of IAcc and IS. IAcc is a more objective measurement, whereas IS is mainly based on the participants’ self-report and individual beliefs.

In recent years, the relevance of interoception became a prominent topic of scientific research, owing to its relationship with different health-related variables (e.g., emotions and stress) and mental disorders [6]. For example, disturbed IAcc and IS were observed in different clinical populations, such as patients with depression, anxiety disorders, obsessive-compulsive disorders (OCD), and eating disorders (bulimia and anorexia nervosa) [7,8,9,10,11,12]. Studies show a positive relationship between the perception and regulation of emotions with interoceptive abilities [13,14]. A mental disorder that is closely related to disturbed emotions, emotional regulation and reduced interoception is depression.

### 1.1. Interoceptive Dimensions and Depression

Different interoceptive dimensions are diminished in depressive patients. Previous studies found a reduced IAcc in depressive patients compared to healthy controls [15,16]. It should be noted that patients with moderate depression showed a decreased IAcc, whereas patients with severe depression did not differ significantly from healthy controls [17]. Focusing on IS, the results also showed a decrease in this variable in depressed patients [7,18,19,20]. For example, depressed patients scored significantly higher on the BPQ and the “Body awareness” subscale, indicating an impaired IS [18]. Another study combined both IS assessments (BPQ and confidence ratings), indicating higher scores on the BPQ and no differences regarding the confidence in depressed patients compared to healthy controls [18]. Specific analysis showed that depression does not facilitate the prediction of IS via BPQ when analysing for sleep quality. Due to the mixed results and different influencing factors (e.g., severe depression, sleep quality, emotion, and decision-making), further research is needed.

### 1.2. Approaches to Change Interoceptive Dimensions

Based on research that demonstrated the depressed patients showed a decrease in interoceptive abilities and their relationship with different health-related variables (e.g., emotional perception and regulation), there is a need to improve these abilities. So far, the results regarding the effectiveness of different approaches (e.g., self-focus-training, biofeedback, power posing, and mindfulness training) in healthy and clinical samples are mixed [21,22,23,24,25]. Concerning IS, findings for the effect of the aforementioned interventions are more consistent, showing an increase in IS in healthy and clinical samples measured via confidence ratings and/or questionnaires [23,26,27,28,29,30]. Some studies examining the effect of cognitive-behavioural therapy (CBT), a well-validated psychosocial and therapeutic intervention to treat mental disorders, regarding interoception have been conducted [8,11,31]. However, only a descriptive trend for the recovery of interoceptive deficits (IAcc and IS) for anorectic patients, during an 8-week CBT course, was observed, whereas patients with OCD did not change in terms of their decreased IAcc-levels [11]. So far, there is no study investigating the effect of CBT on different interoceptive dimensions in depressed patients.

Another opportunity to increase interoceptive dimensions could be mindfulness. This assumption is based on the bodily-focused approach and the specific perception of bodily signals. Mindfulness-Based Stress Reduction (MBSR), conceptualized by Kabat-Zinn [32,33], represents a modern adaptation of old Buddhist mindfulness traditions [34]. The main aspect of this approach includes body scanning, meditation, and mindfulness yoga. While a positive effect of mindfulness training on a depressive sample could be observed [35], studies focusing on healthy samples regarding the effect of mindfulness on interoceptive dimensions found mixed results [23,26,29,36,37,38]. This could be due to the different assessments and the durations of the interventions. Only a single study found an increase in IS through brief mindfulness training in a depressive sample [28]. These results can be confirmed by the findings of an 8-week mindfulness intervention for patients with chronic pain and comorbid depressions [27], or by mindfulness training in women regarding substance use disorder, focussing on IAcc [30].

### 1.3. The Present Study

In conclusion, it appears that specific (therapy) approaches such as CBT and MBSR could be helpful to enhance interoceptive abilities in depressed patients. CBT is a popular and commonly utilized therapeutic approach and has proven to be efficient in depressive disorders. The same can be said about MBSR, which is often used as an additional element during CBT. Because of the beneficial effect of both therapy approaches on interoceptive abilities, deeper insights into CBT’s effect as a stand-alone approach, as well as the effectiveness of a combined approach of CBT and MBSR, are needed. This seems to be necessary because of the increasing number of depressive people. Moreover, the specific approach of mindfulness-based cognitive therapy (MBCT), combining mindfulness and CBT elements, positively affects depression [27]. This supports the relevance of examining the relationship between both techniques more closely. Therefore, the current study aimed to examine the effect of a CBT on clinically depressive patients on both interoceptive dimensions, IAcc and IS, as well as the additional effect of standardized mindfulness training. Consequently, the following hypotheses were formulated:

1. Mindfulness and interoceptive abilities will initially be reduced in the depressed sample compared to healthy controls. Further, these variables will increase in depressed patients over the time course of CBT.
2. In an exploratory manner, the relationship between depressive symptoms, interoceptive abilities and mindfulness in the depressed sample and healthy controls will be examined.
3. Depressed patients, as part of a CBT and an additional MBSR program, will experience increases in their mindfulness level and interoceptive abilities and decreases in their depressive scores over the time course.

## 2. Materials and Methods

### 2.1. Participants

A total of 66 depressed patients were recruited from the psychosomatic clinic in Windach am Ammersee, and received a standardized course of CBT. Out of these, six patients were excluded due to technical issues, resulting in a sample of 60 patients (31 female; age: M = 41.35, SD = 11.2; range: 23–61 years). Based on the semi-structured interviews conducted and the German version of the International Classification of Diseases 10 (ICD-10) [39], all the depressive patients had the diagnosis of a depressive episode (F32) or a recurrent depressive disorder (F33). A group of 39 patients had the diagnosis of moderate and 21 of severe depression.

It is a common occurrence in psychiatric institutions that most patients suffer from comorbidities. In our study, these primarily included burnout (*n* = 20), anxiety disorders (*n* = 14), personality disorders (*n* = 14), somatization disorder (*n* = 9), post-traumatic stress disorder (*n* = 4), and substance abuse (*n* = 4). Most patients had also received (psycho-) pharmacological treatment. Notable medications were Venlafaxine (*n* = 10), Escitalopram (*n* = 9), Mirtazapine (*n* = 8), and Sertraline (*n* = 6).

The CBT concept of the psychosomatic clinic in Windach includes behavioural analysis, which is common in CBT. Therefore, the therapy focused on specific analyses of stress factors and circumstances, and related cognitions, feelings, and actions. Moreover, this therapy aimed to change and decrease the effect of these factors through cognitive–emotional restructuring, behaviour modification, relaxation, and the highlighting of individual strengths and resources. This therapy was complemented by art-, schema-, acceptance- and commitment- as well as body-related therapy and physical activity. Some patients also received an additional 4-week MBSR training course, consisting of two weekly training sessions, including one body scan/week, which, in general should also be trained outside of the therapy setting. Therapists recommended training to the patients who could benefit the most, and also in regard to the overall individual therapeutic concept. We ensured that half of the recruited patients received an additional MBSR training course to investigate the effect of this additional intervention in addition to the CBT (20 of them had a moderate and ten a severe depression).

A total of 52 healthy controls (30 female; age: M = 40.85, SD = 11.19; range: 22–60 years) were recruited via flyers and mail at Ulm University. None of them took medication, had a current psychiatric disorder or severe somatic illness. We attempted to recruit a sample with an equal number of subjects regarding age and gender. Depressed patients received 20 EUR as compensation, whereas healthy controls received 10 EUR.

Considering that there are not many comparable previous studies on depression and interoception, the effect sizes of two previous studies were considered. The first study [11] analysed the relationship of patients with OCD to healthy controls, and the second [8] compared anorectic patients to healthy controls. Both showed a medium to high effect size using Cohen’s criteria [40]. The sample size was calculated using the G*Power 3.1.9 software. Utilizing an effect size of 0.15, specifying an alpha of 0.05, and a power of 0.9 for two groups and measurements resulted in a sample size of *n* = 120. Hence, the number of participants in our study was adequately high for our a priori power analysis.

The groups did not differ in their ages (see Table 1). As hypothesized, depressed patients showed a higher depression level compared to healthy controls at t1. Furthermore, depressed patients indicated significantly lower scores regarding mindfulness, IAcc and IS (confidence and questionnaire) than healthy controls at t1.

### 2.2. Study Design

The study was conducted as per the Declaration of Helsinki. Ethical approval was granted by the Institutional Review Board of Ulm University. All the participants contributed voluntarily and were verbally informed about the aim and design of the study. Furthermore, participants had to sign an informed consent form to take part in the study. Assessment of the depressive patients took place in the psychosomatic clinic in Windach am Ammersee. Healthy controls were assessed in our laboratory at Ulm University. Depressed patients were examined twice, at the beginning (t1) and after four weeks (t2), focusing on the change of the different variables (depression, mindfulness, and interoception) during the CBT period. Additionally, we differentiated between depressed patients, who were only part of the CBT, and patients with CBT and an additional mindfulness training course (CBT + MBSR). In comparison, healthy participants were tested only once. Before the testing session, all the participants were asked to fill out several questionnaires, focusing on depression (Beck Depression Inventory (BDI)) [41], mindfulness (Mindfulness Attention Awareness Scale (MAAS)) [42] and IS via the aforementioned Body awareness subscale of the BPQ [5]. Additionally, the heartbeat tracking task [4], as a behavioural task to assess IAcc, followed by confidence rating to capture another form of IS, was used.

### 2.3. Instruments

The BDI (German version) was used to assess the severity of depression symptoms via self-report [43]. It consists of 21 items with a four-point-rating scale. Total scores range between 0 and 63, whilst lower scores indicate none or minor symptoms and higher scores indicate more severe depressive symptoms. The internal consistency (Cronbach’s alpha) in healthy and clinical populations was good, with a score of 0.89 [44].

The MAAS [42] is a validated self-report questionnaire to measure mindfulness as a trait variable. It consists of 15 Items on a six-point rating scale, ranging from 1 (almost always) to 6 (almost never). It has a good internal consistency in healthy populations with a reported score above Cronbach’s α = 0.80. The German version [45] used in this study also shows a good internal consistency of 0.83 [46].

To measure the IAcc, the heartbeat tracking task [4] was conducted. The BIOPAC^®^ Modell MP35 recorded heartbeats via the BSL PRO Software version 3.7.2. The task consists of four pseudo-randomized intervals (25, 35, 45 and 60 s). The experimenter announced the beginning and the end of each interval. Participants had to perceive their heartbeat. Furthermore, they needed to silently count it and later report the counted heartbeats of the different intervals. Lastly, they were instructed not to use manipulation procedures (e.g., taking their pulse or stopping breathing).

The IAcc was calculated through the formula presented below:

$$IAcc=\frac{1}{4}\sum \left(1-\frac{|recorded\;heartbeats-counted\;heartbeats|}{recorded\;heartbeats}\right)$$

IAcc scores range from 0 to 1, where higher scores indicate a lower discrepancy between the recorded and counted heartbeats, implying a higher heartbeat perception. Former studies in healthy participants reported a mean IAcc score of about 0.69 in healthy samples [8,47,48]. In the study, the internal consistency for IAcc represents excellent scores (Cronbach’s α t1: 0.94; Cronbach’s α t2: 0.95).

To assess the IS, two different methods were considered. First, the IS was measured through confidence ratings. Here, participants needed to report how confident they felt regarding their counted heartbeats during the heartbeat tracking task [4]. Confidence levels were assessed through a Likert scale ranging from 1 = “Absolute insecure about the sensed heartbeat” to 9 = “Completely sure about the sensed heartbeat”. Hereinafter, this variable will be referred to as IS conf. This study found excellent internal consistency scores (Cronbach’s α t1: 0.92.; Cronbach’s α t2: 0.93). Second, IS was assessed through the “Body awareness” subscale of the BPQ [3,5], including 45 items to evaluate the awareness of different bodily sensations, such as muscle tensions, or the temperature of the face, on a five-point scale, ranging from 1 = “Never” to 5 = “Always”. This form of the IS will be described as IS BPQ in the following. Previous studies showed a high internal consistency up to Cronbach’s α = 0.92 [18].

### 2.4. Statistical Analysis

For the analysis, statistical Packages for Social Science (SPSS version 25) were used. Firstly, one-sided Pearson correlation analyses were conducted to investigate the relationship between depression, mindfulness and interoception. A priori contrast analysis was performed using independent sample t-tests (one-tailed) to examine changes in depression, mindfulness, and interoceptive abilities. Further, independent sample *t*-tests (one-tailed) were used to quantify the mean differences of the depressive patients vs. healthy controls regarding the different variables at t1. Lastly, repeated measures ANOVAs (one-tailed) were calculated with the two measurement points (t1 and t2) and conditions (CBT vs. CBT + MBSR). All *p*-values of less than 0.05 were interpreted as statistically significant.

## 3. Results

Depressive symptoms recovered during the four-week CBT course (see Table 2), decreasing from t1 (*M* = 22.25, *SD* = 8.77) to t2 (*M* = 15.58, *SD* = 9.15). Significant results of the independent *t*-test (one-tailed) confirmed this reduction (*t* (59) = 7.79, *p* < 0.001, *d* = 0.74). The depression level was significantly higher for the depressive patients at t1 compared to the healthy controls (*M* = 5.19, *SD* = 4.61), *t* (92.87) = −13.11, *p* < 0.001, *d* = −2.39. These significant differences could be also confirmed for t2, *t* (89.76) = −7.43, *p* < 0.001, *d* = 1.43 (for more details, see Table 2).

**Hypothesis** **1.** *Mindfulness and interoceptive abilities will initially be reduced in depressive patients compared to healthy controls, and these variables will increase over the time course of CBT.*

In line with our hypothesis, the mindfulness level was higher in the healthy sample (*M* = 4.29, *SD* = 0.73) than for depressive patients at t1 (*M* = 3.16, *SD* = 0.81), *t* (110) = 7.75, *p* < 0.001, *d* = 1.47). Similar results could be found for depressive patients at t2, *t* (110) = 4.33, *p* < 0.001, *d* = 0.83. Further, the depressed sample significantly improved its mindfulness level from t1 to t2 (*M* = 3.66, *SD* = 0.81) during CBT, *t* (110) = 7.75, *p* < 0.001, *d* = −1.45 (for more details, see Table 1 and Table 2).

**Figure 1:**
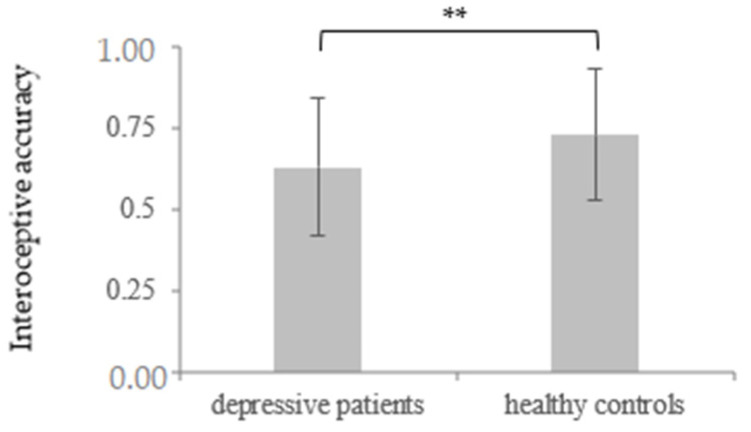
Mean and standard deviation of interoceptive accuracy measured by the heartbeat tracking task regarding depressive patients and healthy controls as a group function at t1. ** *p* < 0.01.

**Figure 2:**
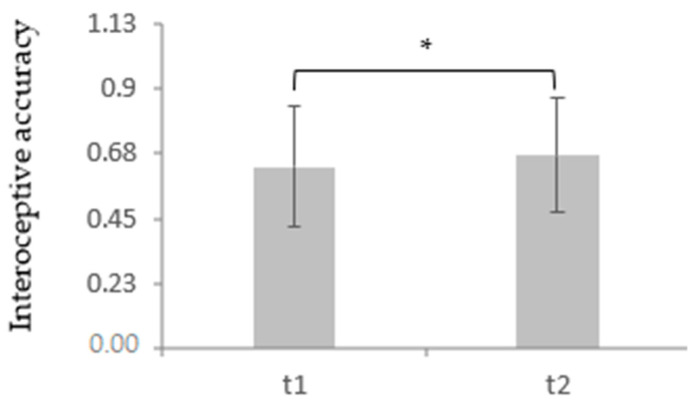
Mean and standard deviation of interoceptive accuracy measured by the heartbeat tracking task over the time course of therapy in a depressed sample. * *p* < 0.05.

At t1, depressive patients (*M* = 0.63, *SD* = 0.21) showed a significantly impaired IAcc in comparison to the healthy controls (*M* = 0.73, *SD* = 0.19), *t* (110) = 2.64, *p* = 0.005, *d* = 0.5. This continued regarding the comparative data from the depressed sample at t2 (*M* = 0.67, *SD* = 0.20) and the healthy controls, *t* (110) = −1.62, *p* = 0.05, *d* = 0.31. Additionally, there was a significant effect of CBT in the depressive sample (*t* (59) = −1.96, *p* = 0.028, *d* = 0.19), showing an improvement of IAcc from t1 to t2. This suggests a general reduction in the IAcc level in the depressed participants over the time course of therapy (for more details, see Table 1 and Table 2 and Figure 1 and Figure 2).

**Figure 3:**
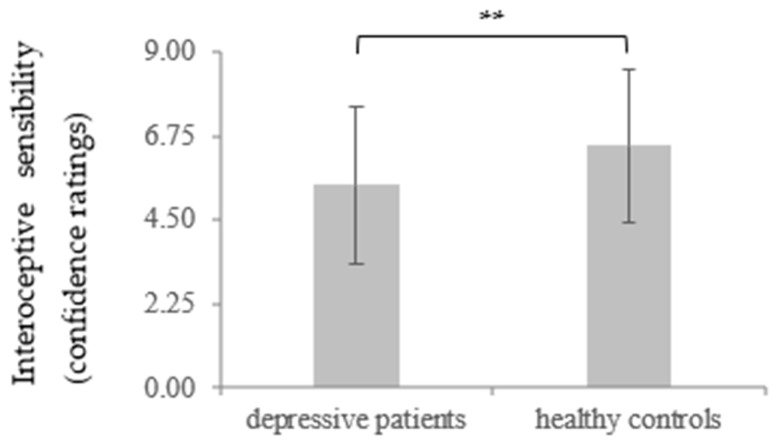
Mean and standard deviation of interoceptive sensibility measured by confidence ratings regarding depressive patients and healthy controls as a group function at t1. ** *p* < 0.01.

**Figure 4:**
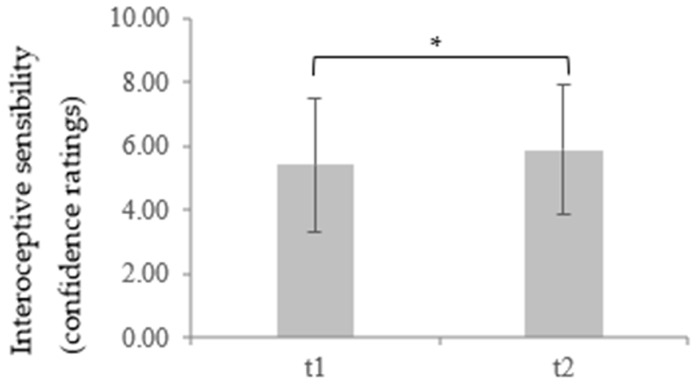
Mean and standard deviation of interoceptive sensibility measured through confidence ratings over the time course of therapy in a depressed sample as a group function. * *p* < 0.05.

Similar to the IAcc, in the analysis of the IS confidence ratings, a significantly lower IAcc between the depressive patients at t1 (*M* = 5.43, *SD* = 2.08) and the healthy controls (*M* = 6.50, *SD* = 2.02) was found, *t* (110) = 2.75, *p* = 0.004, *d* = 0.52. Regarding t2, only a marginally significant difference between the depressed participants (*M* = 5.90, *SD* = 2.05) and healthy controls could be shown, *t*(110) = 1.54, *p* = 0.06, *d* = 0.29. There was also a significant increase in the IS confidence ratings in the depressive patients between t1 and t2 for the IS, *t* (59) = −1.86, *p* = 0.034, *d* = 0.23, indicating a recovery of IS in during time course of therapy (for more details, see Table 1 and Table 4 and Figure 3 and Figure 4).

**Figure 5:**
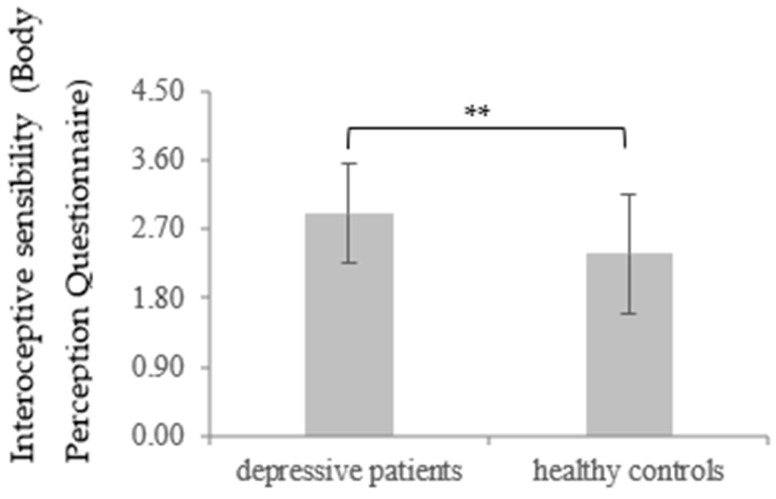
Mean and standard deviation of interoceptive sensibility measured through the “Body awareness” subscale of the Body Perception Questionnaire regarding depressive patients and healthy controls as a group function at t1 (depressive patients vs. healthy controls). ** *p* < 0.01.

**Figure 6:**
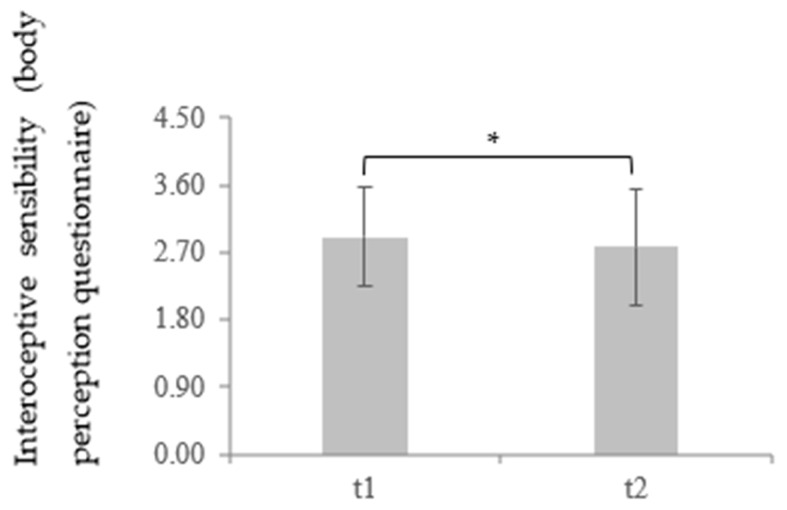
Mean and standard deviation of interoceptive sensibility measured through the “Body awareness” subscale of the Body Perception Questionnaire during the time course of therapy in a depressed sample. * *p* < 0.05.

Lastly, the score of the IS BPQ in depressive patients (*M* = 2.91, *SD* = 0.66) was significantly higher at t1 than for the healthy controls (*M* = 2.38, *SD* = 1.01) *t* (85.97) = −3.23, *p* = 0.001, *d* = 0.63. The significant effect remained for the comparison between data on the depressed sample at t2 (*M* = 2.78, *SD* = 0.78) and the healthy controls, *t* (95.56) = −2.32, *p* = 0.011, *d* = 0.44. Moreover, the score for the IS BPQ regarding the depressive patients decreased significantly between t1 and t2, *t* (59) = 2.16, *p* = 0.018, *d* = 0.18, also showing a recovery of IS during the time course of therapy (for more details, see Table 1 and Table 2 and Figure 5 and Figure 6).

**Hypothesis** **2.** *The relationship between depressive symptoms, interoceptive abilities and mindfulness in the depressed sample, over the time course of CBT, and in healthy controls will be explored.*

In an exploratory manner, we also wanted to analyse the relationship between the different analysed variables. Therefore, Table 3 presents the correlation between depression, mindfulness and interoceptive abilities in depressive patients. Depressive symptoms were significantly negatively correlated with mindfulness (*r* (60) = −0.408, *p* = 0.001) and IAcc (*r* (60) = −0.220, *p* = 0.046) and were positively correlated with IS BPQ (*r* (60) = 0.306, *p* = 0.009) at t1. These relationships could be found again at t2. There was also a positive correlation between IAcc and the IS confidence ratings at t1 (*r* (60) = −0.359, *p* = 0.002) and t2 (*r* (60) = 0.303, *p* = 0.009). Nevertheless, there was no significant correlation of IAcc and the IS confidence ratings with IS BPQ.

Table 4 illustrates the correlation between depression, mindfulness and the different interoceptive abilities. A significant negative correlation between depression and mindfulness (*r* (52) = −0.365, *p* = 0.004) could be found for the healthy controls. Further, a significant positive correlation between mindfulness and the IS confidence ratings (*r* (52) = 0.265, *p* = 0.029), as well as between IAcc and the IS confidence ratings (*r* (52) = 0.338, *p* = 0.017), was observed. No other significant relationships could be found.

**Hypothesis** **3.** *Additional analyses: Comparisons between the effects of CBT and CBT+MBSR on depression, mindfulness and interoceptive abilities, over the time course, will be undertaken.*

Since half of the participants in the depressive group also participated in an additional MBSR training course, an investigation of intra-group effects was conducted to explore possible factors that could have influenced the statistical analysis. Therefore, differences between the CBT and CBT + MBSR conditions, regarding depression, mindfulness and interoception, were analysed via ANOVAs (see Table 5).

The findings regarding the depression level showed a significant main effect of time (*F* (1,58) = 59.63; *p* < 0.001, η^2^ = 0.51). There was no significant main effect of condition (CBT vs. CBT + MBSR; *F* (1,58) = 0.34; *p* = 0.280) or interaction (*F* (1,58) = 0.37; *p* = 0.424), suggesting that the mindfulness trainings of the CBT + MBSR condition had no additional effect on the depression score compared to the stand-alone CBT condition.

Regarding mindfulness, the analyses indicate a significant main effect of time (*F* (1,58) = 29.55; *p* < 0.001, η^2^ = 0.34), and a main effect of condition (*F* (1,58) = 7.35; *p* = 0.004, η^2^ = 0.11). However, the interaction effect was non-significant (*F* (1,58) = 0.003; *p* = 0.429). Against our hypothesis, the additional mindfulness training had no effect on the mindfulness level compared to the CBT alone.

For IAcc, a significant main effect of time (*F* (1,58) = 3.79; *p* = 0.028, η^2^ = 0.06) was found. However, there was no significant difference in relation to the condition (*F* (1,58) = 0.00; *p* = 0.492), nor for the condition x time interaction, (*F* (1,58) = 0.17; *p* = 0.341), meaning that there were no differences between both conditions over time.

For the IS confidence ratings, the results again indicated a main effect of time (*F* (1,58) = 3.40; *p* = 0.035, η^2^ = 0.06) but none were found for the condition (*F* (1,58) = 0.45; *p* = 0.252), or the time x condition interaction (*F* (1,58) = 0.04; *p* = 0.424). These results indicate no differences between both conditions over the time course of therapy.

For IS BPQ, a significant main effect for time (*F* (1,58) = 4.71; *p* = 0.017, η^2^ = 0.06) was found. However, there was no significant main effect of condition (*F* (1,58) = 1.66; *p* = 0.102) and the interaction time x condition (*F* (1,58) = 1.69; *p* = 0.097), meaning that there were no additional effects achieved through the mindfulness training.

## 4. Discussion

This study aimed to evaluate the effect of CBT on different therapy-relevant variables, including depression, mindfulness and interoceptive abilities (IAcc and IS) in a depressive sample compared to healthy controls. Additionally, the potential effect of CBT and an additional MBSR training course, compared to a stand-alone CBT, on these variables in the depressive sample was investigated. In line with our assumptions, depressive patients differed in terms of these variables compared to healthy controls. Additionally, the findings showed a recovery of therapy-relevant variables and interoceptive abilities over the time course of CBT. The IAcc and the confidence rating (IS) significantly increased over the time course of CBT in depressive patients, while the scores in the self-report questionnaire (IS BPQ) significantly decreased. Surprisingly, we could not confirm that patients receiving an additional MBSR training course in addition to CBT improved more in terms of the therapy-relevant variables and interoceptive abilities.

To our knowledge, this is the first study investigating the efficacy of CBT in a depressive sample with an additional focus on interoception. Moreover, previous studies investigating the recovery of these variables during the time course of CBT in an anorectic clinical sample and those with OCD could not confirm these findings [8,11]. In comparison, we only included two measurement points over four weeks, whereas the other studies comprised three measurement points over 8–12 weeks, depending on the therapy. It should be noted that the former studies showed the highest improvement of interoceptive abilities between t1 and t2, which is comparable with the presented 4-week design of this study [8]. To conclude, we have no evidence in favour of a more extended period of CBT in terms of the efficacy of the investigated variables.

Moreover, it should be noted that a part of this depressive sample received additional mindfulness training, which could have influenced the results in a positive direction. In previous studies, an effect of mindfulness training in depressed patients on IS measured through questionnaires (the Multidimensional Assessment of Interoceptive Awareness (MAIA), [49]) could be found [27,28]. The positive effect of mindfulness on interoception is reasonable because of its body-oriented effect [50]. Previous studies pointed out that a non-significant effect in the clinical samples could be based on the missing focus, within CBT, on interoceptive abilities and the body, which would undermine our assumption of the positive effect from the mindfulness intervention [8,11]. Additionally, the positive results regarding interoceptive abilities due to CBT can be underpinned by comparing depressed patients and healthy controls, indicating that depressed patients differ significantly in terms of their therapy-relevant variables and interoceptive abilities, which is in line with previous studies [7,15,16,18]. Nevertheless, all variables recovered in the timeline and became closer to healthy control data. It is worth noting that we could only present data from healthy controls at one point of measurement.

The exploratory correlation analyses showed a negative relationship between depression and mindfulness and most of the interoceptive abilities in the depressive sample. The negative relationship between depression and mindfulness follows previous research conducted with depressive and healthy samples [47,51,52,53]. Only one study investigated the relationship between depression and interoception, finding a negative relationship between depressive symptoms and IAcc in a student sample [54]. Consequently, the findings are in line for the depressive sample but not for the healthy sample. It should be noted that the healthy sample in the aforementioned study differs in comparison to our study. While participants there showed only minimal or low scores in depression (3.3%), our study depicts 13.5% of participants as having depressive symptoms in this quantile. This could be a possible explanation for the differences in both studies. Notably, we could not confirm the positive relationship between mindfulness and interoceptive abilities in the clinical sample. Similar results could be observed for mindfulness and IAcc in healthy participants [23]. Nevertheless, they showed a positive relationship between mindfulness and confidence for a healthy sample, which is in line with our correlation analyses of the healthy sample, but not with the depressed patients. Lastly, in the healthy sample, only a negative association could be found for depression and mindfulness and a positive relationship between mindfulness and confidence. Comparable results were also found in other studies, as noted above [23,47,51,52,53].

The last hypothesis expected that CBT combined with an additional MBSR training course would be more effective than a stand-alone CBT course in terms of the effects on depressive symptoms, mindfulness, and interoceptive abilities. The results indicate that both groups recovered as well during the time course of the CBT period as with the combination of CBT and MBSR training. However, patients with the additional MBSR training did not differ significantly from patients with the stand-alone CBT regarding therapy-relevant variables and interoceptive abilities. Until now, only a few studies [27,28] indicate an improvement of IS through mindfulness training in a depressive (comorbid) sample. It should be noted that there are methodological differences. In the study of de Jong and colleagues [27], the standardized 8-week MBCT was compared to a treatment-as-usual condition [27]. In comparison, Fissler et al. [28] used an unstandardized brief mindfulness training. The intervention in this study is a standardized MBSR training, but in line with the duration of the CBT course and the later beginning of the mindfulness training, it was shortened to a 4-week training period. The participants received the mindfulness training for four weeks and were additionally asked to integrate the exercises into their everyday lives in addition to other therapeutic elements. Nonetheless, in the official MBSR training system [32,33], an eight-week program is proposed.

Consequently, the differences in standardization and duration could have caused different results in this and recent studies. This assumption is supported by a study examining the effect of a body scan after 8-weeks [50]. Specifically, post-hoc analyses indicated an improvement in interoceptive abilities between t2 (four weeks) and t3 (eight weeks) as well as between t1 (beginning) and t3. Consequently, longer durations of interventions are needed to affect interoceptive abilities (IAcc and IS). Moreover, the aforementioned studies [27,28] used the MAIA to measure IS, whereas the presented study used the “Body awareness” subscale of the BPQ. This subscale mainly focuses on the general perception of bodily symptoms, whereas the MAIA concentrates on a broader range of interoception and related variables (e.g., noticing, attention regulation, emotional awareness, and self-regulation), leading to different findings. Depression is closely related to somatic symptoms such as impaired sleep, pain, and different somatic concerns, which can also be seen as an impairing factor regarding depressive symptoms [55]. Previous studies found a positive relation between somatic symptoms, depression, and the BPQ [56,57], indicating that the BPQ takes somatic aspects into account. Therefore, using the BPQ seems to be beneficial in studies focussing on depression.

To sum up, based on former studies indicating that interoceptive abilities can improve in healthy and clinical samples, there is a need for longer interventions (minimum eight weeks). Additionally, an investigation of other interoceptive modalities could be interesting. For example, the respiratory system might be highly relevant because of the higher focus on the breath found in different mindfulness exercises. One study [37] considered the IAcc via a respiratory discrimination task, indicating no effect of a 3-week mindfulness app intervention. Nonetheless, based on our above idea, this could be explained by the short duration of the intervention.

### Limitations

The presented study has some limitations regarding the analyses of the different conditions (CBT vs. CBT and MBSR), which should be considered. As is typical in psychiatric and psychosomatic clinics, patients profit from a variety of therapeutic techniques and approaches. Hence, overlapping effects on the same variable, such as interoception from different approaches, are common. For example, psychotherapists working under the stand-alone CBT condition could use mindfulness-based techniques on an everyday basis. This could also explain the increase in mindfulness scores that was found to be associated with this condition. Moreover, this could also be the reason why we did not find any differences between the conditions. Further, the quasi-experimental design of this study must be pointed out. Therapists chose and recommended a different specific combination of therapeutic offers of the clinic for every single patient so that the patient profited most, which was also the case for the additional MBSR training course in addition to the CBT. Consequently, randomization between the two groups was not possible. Therefore, the presented analyses should be seen as an initial approach to identify the effects of a supplementary mindfulness training course on interoception in depressive patients. More specific training or additive modules should be considered in future studies and therapy settings. For example, biofeedback training focusing on cardiac perception [22], or mindfulness training with a high focus on body-oriented modules, could be helpful.

## 5. Conclusions

Our study replicates previous findings, pointing out that interoception is decreased in depression and that these deficits recover during the time course of CBT [6,58]. Further research is needed to evaluate the long-term development of interoceptive abilities. It could be beneficial to investigate the research question of whether decreased interoceptive abilities are a risk factor for relapse or mental disorders in general. Training of interoceptive abilities appears to be a fundamental approach for further research regarding mental illness and should be further elaborated in future research.

## Figures and Tables

**Table 1:** Descriptive variables of depressive patients and healthy controls at t1.

	Depressive Patients (*n* = 60)		Healthy Controls (*n* = 52)		t(df)	*p*	*d*
	M	SD	M	SD			
Age	41.35	11.16	40.85	11.19	−24(110)	0.406	−0.05
BDI	22.25	8.77	5.19	4.61	−13.11(91.87)	0.001	−2.39
MAAS	3.16	0.81	4.29	0.73	7.75(110)	0.001	1.45
IAcc	0.63	0.21	0.73	0.19	2.64(110)	0.005	0.50
IS conf	5.43	2.08	6.49	2.02	2.75(110)	0.004	0.52
IS BPQ	2.91	0.66	2.38	1.01	−3.23(85.97)	0.001	−0.63

Note: *M* = mean; *SD* = standard deviation; *d* = Cohen’s *d*; one-tailed *t*-test; BDI = Beck Depression Inventory; MAAS = Mindfulness Attention Awareness Scale; IAcc = Interoceptive Accuracy; IS conf = Interoceptive Sensibility (confidence rating); IS BPQ = Interoceptive Sensibility measured through the Body Perception Questionnaire.

**Table 2:** Descriptive variables for the depressive patients and healthy controls at different measurement points.

	Depressive Patients (*n* = 60)				Healthy Controls (*n* = 52)	
	t1		t2			
	*M*	*SD*	*M*	*SD*	*M*	*SD*
BDI	22.25	8.77	15.58	9.15	5.19	4.61
MAAS	3.16	0.81	3.66	0.81	4.29	0.73
IAcc	0.63	0.21	0.67	0.20	0.73	0.19
IS conf	5.43	2.08	5.90	2.05	6.50	2.02
IS BPQ	2.91	0.66	2.78	0.78	2.38	1.01

*Note*. *M* = mean; *SD* = standard deviation; BDI = Beck Depression Inventory; MAAS = Mindful Attention Awareness Scale; IAcc = Interoceptive Accuracy; IS conf = Interoceptive Sensibility (confidence rating); IS BPQ = Interoceptive Sensibility measured through the Body Perception Questionnaire.

**Table 3:** Pearson’s correlation analyses focusing on depression, mindfulness, and the different interoceptive abilities at t1 and t2 in depressive patients.

Variable	1	2	3	4	5	6	7	8	9	10
1. BDI t1	1	0.727 **	−0.408 **	−0.473 **	−0.220 *	−0.034	−0.082	−0.253 *	0.306 *	0.299 *
2. BDI t2		1	−0.417 **	−0.614 **	−0.209	−0.008	−0.257 *	−0.195	0.215 *	0.351 **
3. MAAS t1			1	0.622 **	0.007	−0.137	−0.038	−0.158	−0.143	−0.219 *
4. MAAS t2				1	−0.023	−0.094	−0.019	0.087	−0.154	0.318 **
5. IAcc t1					1	0.678 **	0.359 **	0.349 **	−0.114	0.186
6. IAcc t2						1	0.061	0.303 **	−0.110	0.152
7. IS conf t1							1	0.541 **	0.117	−0.048
8. IS conf t2								1	0.005	−0.049
9. IS BPQ t1									1	0.806 **
10. IS BPQ t2										1

*Note*: BDI = Beck Depression Inventory; MAAS = Mindful Attention Awareness Scale; IAcc = Interoceptive Accuracy; IS conf = Interoceptive Sensibility (confidence rating); IS BPQ = Interoceptive Sensibility (Body Perception Questionnaire). * *p* < 0.05, ** *p* < 0.01.

**Table 4:** Pearson’s correlation analyses focusing on depression, mindfulness and the different interoceptive abilities in healthy controls.

Variable	1	2	3	4	5
1. BDI	1	−0.365 **	−0.112	−0.014	0.080
2. MAAS		1	−0.079	0.265 *	0.100
3. IAcc			1	0.338 **	0.028
4. IS conf				1	0.047
5. IS BPQ					1

*Note:* BDI = Beck Depression Inventory; MAAS = Mindful Attention Awareness Scale; IAcc = Interoceptive Accuracy; IS conf = Interoceptive Sensibility (confidence rating); IS BPQ = Interoceptive sensibility (Body Perception Questionnaire). * *p* < 0.05, ** *p* < 0.01.

**Table 5:** Differences between depression, mindfulness and the different interoceptive abilities in the different conditions (CBT vs. CBT + MBSR) at t1 and t2.

	CBT (*n* = 30)		CBT + MBSR (*n* = 30)		*t* (df = 58)	*p*	*d*
	*M*	*SD*	*M*	*SD*			
BDI t1	21.70	9.09	22.80	8.56	−0.48	0.315	−0.13
BDI t2	14.87	9.03	16.30	9.37	−0.60	0.275	−0.16
MAAS t1	3.41	0.68	2.91	0.86	2.51	0.008	0.65
MAAS t2	3.89	0.87	3.42	0.69	2.32	0.012	0.60
IAcc t1	0.62	0.23	0.63	0.20	−0.14	0.444	−0.04
IAcc t2	0.67	0.21	0.66	0.19	0.19	0.424	0.05
IS conf t1	5.56	2.13	5.29	2.05	0.49	0.312	0.13
IS conf t2	6.08	1.85	5.72	2.25	0.69	0.247	0.18
IS BPQ t1	3.06	0.72	2.75	0.57	1.81	0.038	0.47
IS BPQ t2	2.85	0.91	2.70	0.63	0.74	0.232	0.19

*Note*. *M* = mean; *SD* = standard deviation; *d* = Cohen’s *d*; one-tailed *t*-test; CBT = Cognitive-behavioural Therapy; MBSR = Mindfulness-based Stress Reduction; BDI = Beck Depression Inventory; MAAS = Mindful Attention Awareness Scale; MBSR = Mindfulness-based Stress Reduction; CBT = Cognitive-behavioural Therapy; IAcc = Interoceptive Accuracy; IS conf = Interoceptive Sensibility (confidence rating); IS BPQ = Interoceptive Sensibility measured through the Body Perception Questionnaire.

## Data Availability

The data presented in this study are available on request from the corresponding author.
